# Glossary of terms: A shared understanding of the common terms used to describe psychological trauma, version 3.0

**DOI:** 10.24095/hpcdp.44.9.09

**Published:** 2024-09

This corrigendum is being published to acknowledge the contribution of Dr. Deborah Norris, which was omitted from the original version of the article: 

Heber A, Testa V, Groll D, Ritchie K, Tam-Seto L, Mulligan A, Sullo E, Schick A, Bose E, Jabbari Y, Lopes J, Carleton RN. Glossary of terms: A shared understanding of the common terms used to describe psychological trauma, version 3.0. Health Promot Chronic Dis Prev Can. 2023;43(10/11). https://doi.org/10.24095/hpcdp.43.10/11.09

## Before correction

***Acknowledgements***

We thank the following partner organizations (in alphabetical order) for their support in making this resource a reality: the Atlas Institute for Veterans and Families, the Canadian Institute for Military and Veteran Health Research (CIMVHR), the Canadian Institute for Pandemic Health Education and Response (CIPHER), the Canadian Institute for Public Safety Research and Treatment (CIPSRT), Department of National Defence Terminology Board, McMaster University, the Public Health Agency of Canada, Queen’s University, and Veterans Affairs Canada. In addition, we would like to thank our contributors’ employers and universities who allowed their members the time needed to contribute to this glossary.

The senior authors team wishes to acknowledge all the people with lived experience and other stakeholders who dedicated their time and provided thoughtful contributions in collaboratively defining military sexual trauma.

The senior authors team is grateful to the following individuals for their contributions to the development of this glossary (contributors are listed alphabetically by last name):

LCol (Ret’d) Suzanne Bailey, Curriculum Development Lead, Road to Mental Readiness, Canadian Forces Health Services Group, Department of National Defence

Dr. Mary Bartram, PhD, RSW, Director, Policy, Mental Health Commission of Canada, Adjunct Research Professor, School of Public Policy and Administration, Carleton University

Dr. Suzette Brmault-Phillips, OT, PhD, DCA, Associate Professor, Department of Occupational Therapy, Faculty of Rehabilitation Medicine, University of Alberta; Director, HiMARC (Heroes in Mind, Advocacy and Research Consortium), University of Alberta

Dr. Lori Buchart, PhD, DBA, Co-Founder and Former Co-Chair, It’s Not Just 20K (INJ20K, formerly It’s Not Just 700)

Col Marilynn S. Chenette, CD, CHE, Deputy Commander, Health Services Division (HS Div), Canadian Armed Forces

Dr. Heidi Cramm, PhD, OT Reg. (Ont.), Professor, School of Rehabilitation Therapy, Queen’s University

Department of National Defence Terminology Board

Michelle Douglas, Executive Director, LGBT Purge Fund

Dr. Susan Dowler, PhD, CPsych, Chief Clinical Psychologist, Canadian Forces Health Services Group, Department of National Defence

A/Superintendent Lorraine Downey, BBA, Operations, Peer Support Coordinator, Ottawa Paramedic Service

Gabrielle Dupuis, MSc, Director, Research Partnerships, Atlas Institute for Veterans and Families

Dr. Maya Eichler, PhD, Associate Professor, Political and Canadian Studies, Women’s Studies, Mount Saint Vincent University; Canada Research Chair in Social Innovation and Community Engagement and leader of the Centre for Social Innovation and Community Engagement in Military Affairs, Mount Saint Vincent University

Dr. Murray Enns, MD, FRCPC, Professor, Department of Psychiatry, University of Manitoba; Medical Director and Staff Psychiatrist, Operational Stress Injury Clinic; Staff Psychiatrist, Adult, Health Sciences Centre; Adjunct Scientist, Manitoba Centre for Health Policy

Dr. Kyle Handley, Clinical Psychologist, York Regional Police, Chair of the Canadian Association Chiefs of Police Psychological Services Committee

Dr. Marnin J. Heisel, PhD, CPsych, Professor, Departments of Psychiatry and of Epidemiology & Biostatistics, University of Western Ontario; Scientist, Lawson Health Research Institute

Christine Hutchins, Senior Director, Office of Women and LGBTQ2 Veterans, Veterans Affairs Canada

Dr. Ruth Lanius, MD, PhD, FRCPC, Professor, Department of Psychiatry; Director of Posttraumatic Stress Disorder research unit; Harris-Woodman Chair in Psyche and Soma, Schulich School of Medicine & Dentistry, Western University; Collaborating Clinical Scientist, Homewood Research Institute

Dr. Vivien Lee, PhD, CPsych, Chief Psychologist, Commander, Healthy Workplace Team, Ontario Provincial Police; Clinical Advisor, Boots on the Ground

Sgt. Brent MacIntyre, BA (Hons.) Criminology, BA Sociology, Respect, Ethics and Values, Ottawa Police Service

Dr. Randi McCabe, PhD, CPsych, Professor, Department of Psychiatry & Behavioural Neurosciences, McMaster University; Co-Founder, Hamilton Centre for Cognitive Behavioural Therapy

Dr. Megan McElheran, RPsych, Clinical Psychologist, Wayfound Mental Health Group

Dr. Margaret McKinnon, PhD, CPsych, Homewood Chair in Mental Health and Trauma, Professor and Associate Chair, Research, Department of Psychiatry and Behavioural Neurosciences, McMaster University; Research Lead, Mental Health & Addictions Services, St. Joseph’s Healthcare Hamilton; Senior Scientist, Homewood Research Institute

Dr. Anthony Nazarov, PhD, PMP, Associate Scientific Director, MacDonald Franklin OSI Research Centre; Research Scientist and Adjunct Research Professor, Department of Psychiatry, Schulich School of Medicine & Dentistry, Western University; Adjunct Scientist, Lawson Health Research Institute; Adjunct Research Professor, Department of Psychiatry and Behavioural Neurosciences, McMaster University

Dr. Andrew Nicholson, PhD, Assistant Professor, School of Psychology, University of Ottawa; Director of Clinical Research, Atlas Institute for Veterans and Families

Lt Scott Patey, OFS Peer Support Co-Coordinator, Ottawa Fire Services

Alain Pellegroms, Training Coordinator, OFS Medical Program Coordinator, OFS Peer Support Coordinator, Training Division, Ottawa Fire Service

Dr. Jill Price, PhD, Postdoctoral Fellow, Canadian Institute for Public Safety Research and Treatment (CIPSRT); Sessional Professor, Campion College, University of Regina

Dr. Rosemary Ricciardelli, PhD, Professor and Research Chair in Safety, Security, and Wellness, School of Maritimes Studies, Fisheries and Marine Institute, Memorial University of Newfoundland

Dr. J. Don Richardson, MD, FRCPC, Consultant Psychiatrist, Medical Director, St. Joseph’s Health Care London’s Operational Stress Injury Clinic; Scientific Director, MacDonald Franklin OSI Research Centre; Medical Advisor, Atlas Institute for Veterans and Families; Professor and Wellness Lead, Tanna Schulich Chair in Neuroscience and Mental Health, Schulich School of Medicine & Dentistry, Western University; Associate Scientist, Lawson Health Research Institute

Dr. Maya Roth, PhD, CPsych, Clinical Psychologist, St. Joseph’s Healthcare London’s Operational Stress Injury Clinic; Affiliated Scientist, MacDonald Franklin OSI Research Centre; Associate Member, Yeates School of Graduate Studies, Toronto Metropolitan University; Adjunct Clinical Professor, Schulich School of Medicine and Dentistry, Western University; Associate Scientist, Lawson Health Research Institute

Martine Roy, Chair, Board of Directors, LGBT Purge Fund

Capt Sam Samplonius, Co-Founder, INJ20K

PC Benny Seto, BBE, Mobile Crisis Intervention Team, Toronto Police Service

Dr. Norman Shields, PhD, CPsych, National Chief Psychologist, Occupational Health and Safety Branch, Royal Canadian Mounted Police

Dr. Lorraine Smith-MacDonald, PhD, MA, MDiv, CCC, Postdoctoral Fellow, Heroes in Mind, Advocacy, and Research Consortium (HiMARC), Faculty of Rehabilitation Medicine, University of Alberta

Dr. Heather Stuart, PhD, FRSC, CM, Professor, Department of Public Health Sciences, Department of Psychiatry and the School of Rehabilitation Therapy, Queen’s University; Bell Canada Chair Mental Health and Anti-Stigma Research, Queen’s University

LCol Dr. Andrea Tuka, CD, MD, FRCPC, Clinical Assistant Professor, Department of Psychiatry, University of British Columbia; Chief of Psychiatry, Canadian Armed Forces

## After correction

***Acknowledgements***

We thank the following partner organizations (in alphabetical order) for their support in making this resource a reality: the Atlas Institute for Veterans and Families, the Canadian Institute for Military and Veteran Health Research (CIMVHR), the Canadian Institute for Pandemic Health Education and Response (CIPHER), the Canadian Institute for Public Safety Research and Treatment (CIPSRT), Department of National Defence Terminology Board, McMaster University, the Public Health Agency of Canada, Queen’s University, and Veterans Affairs Canada. In addition, we would like to thank our contributors’ employers and universities who allowed their members the time needed to contribute to this glossary.

The senior authors team wishes to acknowledge all the people with lived experience and other stakeholders who dedicated their time and provided thoughtful contributions in collaboratively defining military sexual trauma.

The senior authors team is grateful to the following individuals for their contributions to the development of this glossary (contributors are listed alphabetically by last name):

LCol (Ret’d) Suzanne Bailey, Curriculum Development Lead, Road to Mental Readiness, Canadian Forces Health Services Group, Department of National Defence

Dr. Mary Bartram, PhD, RSW, Director, Policy, Mental Health Commission of Canada, Adjunct Research Professor, School of Public Policy and Administration, Carleton University

Dr. Suzette Brmault-Phillips, OT, PhD, DCA, Associate Professor, Department of Occupational Therapy, Faculty of Rehabilitation Medicine, University of Alberta; Director, HiMARC (Heroes in Mind, Advocacy and Research Consortium), University of Alberta

Dr. Lori Buchart, PhD, DBA, Co-Founder and Former Co-Chair, It’s Not Just 20K (INJ20K, formerly It’s Not Just 700)

Col Marilynn S. Chenette, CD, CHE, Deputy Commander, Health Services Division (HS Div), Canadian Armed Forces

Dr. Heidi Cramm, PhD, OT Reg. (Ont.), Professor, School of Rehabilitation Therapy, Queen’s University

Department of National Defence Terminology Board

Michelle Douglas, Executive Director, LGBT Purge Fund

Dr. Susan Dowler, PhD, CPsych, Chief Clinical Psychologist, Canadian Forces Health Services Group, Department of National Defence

A/Superintendent Lorraine Downey, BBA, Operations, Peer Support Coordinator, Ottawa Paramedic Service

Gabrielle Dupuis, MSc, Director, Research Partnerships, Atlas Institute for Veterans and Families

Dr. Maya Eichler, PhD, Associate Professor, Political and Canadian Studies, Women’s Studies, Mount Saint Vincent University; Canada Research Chair in Social Innovation and Community Engagement and leader of the Centre for Social Innovation and Community Engagement in Military Affairs, Mount Saint Vincent University

Dr. Murray Enns, MD, FRCPC, Professor, Department of Psychiatry, University of Manitoba; Medical Director and Staff Psychiatrist, Operational Stress Injury Clinic; Staff Psychiatrist, Adult, Health Sciences Centre; Adjunct Scientist, Manitoba Centre for Health Policy

Dr. Kyle Handley, Clinical Psychologist, York Regional Police, Chair of the Canadian Association Chiefs of Police Psychological Services Committee

Dr. Marnin J. Heisel, PhD, CPsych, Professor, Departments of Psychiatry and of Epidemiology & Biostatistics, University of Western Ontario; Scientist, Lawson Health Research Institute

Christine Hutchins, Senior Director, Office of Women and LGBTQ2 Veterans, Veterans Affairs Canada

Dr. Ruth Lanius, MD, PhD, FRCPC, Professor, Department of Psychiatry; Director of Posttraumatic Stress Disorder research unit; Harris-Woodman Chair in Psyche and Soma, Schulich School of Medicine & Dentistry, Western University; Collaborating Clinical Scientist, Homewood Research Institute

Dr. Vivien Lee, PhD, CPsych, Chief Psychologist, Commander, Healthy Workplace Team, Ontario Provincial Police; Clinical Advisor, Boots on the Ground

Sgt. Brent MacIntyre, BA (Hons.) Criminology, BA Sociology, Respect, Ethics and Values, Ottawa Police Service

Dr. Randi McCabe, PhD, CPsych, Professor, Department of Psychiatry & Behavioural Neurosciences, McMaster University; Co-Founder, Hamilton Centre for Cognitive Behavioural Therapy

Dr. Megan McElheran, RPsych, Clinical Psychologist, Wayfound Mental Health Group

Dr. Margaret McKinnon, PhD, CPsych, Homewood Chair in Mental Health and Trauma, Professor and Associate Chair, Research, Department of Psychiatry and Behavioural Neurosciences, McMaster University; Research Lead, Mental Health & Addictions Services, St. Joseph’s Healthcare Hamilton; Senior Scientist, Homewood Research Institute

Dr. Anthony Nazarov, PhD, PMP, Associate Scientific Director, MacDonald Franklin OSI Research Centre; Research Scientist and Adjunct Research Professor, Department of Psychiatry, Schulich School of Medicine & Dentistry, Western University; Adjunct Scientist, Lawson Health Research Institute; Adjunct Research Professor, Department of Psychiatry and Behavioural Neurosciences, McMaster University

Dr. Andrew Nicholson, PhD, Assistant Professor, School of Psychology, University of Ottawa; Director of Clinical Research, Atlas Institute for Veterans and Families

Dr. Deborah Norris, PhD, Professor, Department of Family Studies & Gerontology, Mount Saint Vincent University

Lt Scott Patey, OFS Peer Support Co-Coordinator, Ottawa Fire Services

Alain Pellegroms, Training Coordinator, OFS Medical Program Coordinator, OFS Peer Support Coordinator, Training Division, Ottawa Fire Service

Dr. Jill Price, PhD, Postdoctoral Fellow, Canadian Institute for Public Safety Research and Treatment (CIPSRT); Sessional Professor, Campion College, University of Regina

Dr. Rosemary Ricciardelli, PhD, Professor and Research Chair in Safety, Security, and Wellness, School of Maritimes Studies, Fisheries and Marine Institute, Memorial University of Newfoundland

Dr. J. Don Richardson, MD, FRCPC, Consultant Psychiatrist, Medical Director, St. Joseph’s Health Care London’s Operational Stress Injury Clinic; Scientific Director, MacDonald Franklin OSI Research Centre; Medical Advisor, Atlas Institute for Veterans and Families; Professor and Wellness Lead, Tanna Schulich Chair in Neuroscience and Mental Health, Schulich School of Medicine & Dentistry, Western University; Associate Scientist, Lawson Health Research Institute

Dr. Maya Roth, PhD, CPsych, Clinical Psychologist, St. Joseph’s Healthcare London’s Operational Stress Injury Clinic; Affiliated Scientist, MacDonald Franklin OSI Research Centre; Associate Member, Yeates School of Graduate Studies, Toronto Metropolitan University; Adjunct Clinical Professor, Schulich School of Medicine and Dentistry, Western University; Associate Scientist, Lawson Health Research Institute

Martine Roy, Chair, Board of Directors, LGBT Purge Fund

Capt Sam Samplonius, Co-Founder, INJ20K

PC Benny Seto, BBE, Mobile Crisis Intervention Team, Toronto Police Service

Dr. Norman Shields, PhD, CPsych, National Chief Psychologist, Occupational Health and Safety Branch, Royal Canadian Mounted Police

Dr. Lorraine Smith-MacDonald, PhD, MA, MDiv, CCC, Postdoctoral Fellow, Heroes in Mind, Advocacy, and Research Consortium (HiMARC), Faculty of Rehabilitation Medicine, University of Alberta

Dr. Heather Stuart, PhD, FRSC, CM, Professor, Department of Public Health Sciences, Department of Psychiatry and the School of Rehabilitation Therapy, Queen’s University; Bell Canada Chair Mental Health and Anti-Stigma Research, Queen’s University

LCol Dr. Andrea Tuka, CD, MD, FRCPC, Clinical Assistant Professor, Department of Psychiatry, University of British Columbia; Chief of Psychiatry, Canadian Armed Forces

The original online version of the article was modified on September 11, 2024, to reflect this change. The PDF version of the article reflects the updated version of the Acknowledgements.

